# Cross-Sectoral Zoonotic Disease Surveillance in Western Kenya: Identifying Drivers and Barriers Within a Resource Constrained Setting

**DOI:** 10.3389/fvets.2021.658454

**Published:** 2021-06-08

**Authors:** Lian Francesca Thomas, Jonathan Rushton, Salome A. Bukachi, Laura C. Falzon, Olivia Howland, Eric M. Fèvre

**Affiliations:** ^1^Institute of Infection, Veterinary and Ecological Sciences, University of Liverpool, Leahurst Campus, Neston, United Kingdom; ^2^International Livestock Research Institute, Nairobi, Kenya; ^3^Centre of Excellence for Sustainable Food Systems, University of Liverpool, Liverpool, United Kingdom; ^4^Institute of Anthropology, Gender & African Studies, University of Nairobi, Nairobi, Kenya

**Keywords:** one health, surveillance, resource allocation, prioritisation, livestock, zoonoses, Kenya

## Abstract

**Background:** Collaboration between the human and animal health sectors, including the sharing of disease surveillance data, has the potential to improve public health outcomes through the rapid detection of zoonotic disease events prior to widespread transmission in humans. Kenya has been at the forefront of embracing a collaborative approach in Africa with the inception of the Zoonotic Disease Unit in 2011. Joint outbreak responses have been coordinated at the national level, yet little is currently documented on cross-sectoral collaboration at the sub-national level.

**Methods:** Key informant interviews were conducted with 28 disease surveillance officers from the human and animal health sectors in three counties in western Kenya. An inductive process of thematic analysis was used to identify themes relating to barriers and drivers for cross-sectoral collaboration.

**Results:** The study identified four interlinking themes related to drivers and barriers for cross-sectoral collaboration. To drive collaboration at the sub-national level there needs to be a clear identification of “common objectives,” as currently exemplified by the response to suspected rabies and anthrax cases and routine meat hygiene activities. The action of collaboration, be it integrated responses to outbreaks or communication and data sharing, require “operational structures” to facilitate them, including the formalisation of reporting lines, supporting legislation and the physical infrastructure, from lab equipment to mobile phones, to facilitate the activities. These structures in turn require “appropriate resources” to support them, which will be allocated based on the “political will” of those who control the resources.

**Conclusions:** Ongoing collaborations between human and animal disease surveillance officers at the sub-national level were identified, driven by common objectives such as routine meat hygiene and response to suspected rabies and anthrax cases. In these areas a suitable operational structure is present, including a supportive legislative framework and clearly designated roles for officers within both sectors. There was support from disease surveillance officers to increase their collaboration, communication and data sharing across sectors, yet this is currently hindered by the lack of these formal operational structures and poor allocation of resources to disease surveillance. It was acknowledged that improving this resource allocation will require political will at the sub-national, national and international levels.

## Introduction

Global awareness of zoonotic disease emergence and the risks these pose both to human health and our global economy has been growing steadily over the last two decades and has been thrown into sharp relief by the ongoing COVID-19 pandemic. Robust, disease surveillance systems are integral to the prevention and control of zoonoses and it has been proposed that many benefits may arise from collaboration between, or even the integration of, surveillance activities across the animal and human health sector. Identifying zoonoses within the animal host prior to transmission to or between humans has the potential to mitigate outbreaks at source, saving lives and potentially large economic burdens (1). The mobilisation of cross-sectoral response teams in the face of an outbreak or in a case investigation allows for operational cost-sharing and can enhance capacity strengthening providing cross-sectoral learning opportunities (2). It is also hypothesised that sharing of facilities, such as laboratories, will enhance the cost-effectiveness of surveillance activities and assist in the retention of laboratory skills by ensuring laboratories work at optimal capacity (3). Routine surveillance activities within humans, animals and food products, with interoperable data sharing between sectors, can assist in the monitoring for presence and trends of pathogen occurrence and identification of risk factors, allowing for appropriate allocation of resources to mitigate the burden of zoonoses and foodborne disease (4, 5).

Such cross-sector collaboration is a key component of the “One Health” (OH) concept, which has been widely championed by the international community and is seen as integral to the success of the Global Health Security Agenda (GHSA) (6–9). Indeed, technical agencies of the United Nations, most notably the World Health Organization (WHO), Food & Agriculture Organization (FAO) and UN Environment Program (UNEP), together with the World Organization for Animal Health (OIE) (the “tripartite plus”), are working together to strengthen OH working at the international level, such as through the Global Early Warning & Response System and through support for the development of national networks (10). Regional bodies, such as Africa CDC and the African Union have also embraced the OH concept to guide their activities and several regional networks have been convened to build capacity and support OH working (11, 12).

Kenya has been proactive in adopting the concepts of OH, with the establishment in 2011 of one of the first dedicated national offices, the Zoonotic Disease Unit (ZDU) (13). The mission of this unit, which sits between the Ministry of Health (MoH) and the Ministry of Agriculture, Livestock & Fisheries (MALF), is to establish and maintain collaboration at the animal, human, ecosystem interface for the prevention and control of zoonotic diseases (14). In line with the GHSA, Kenya has undertaken a prioritisation exercise for zoonoses, has developed a national action plan for Antimicrobial Resistance and a national strategy for elimination of dog-mediated rabies (15–18).

Under the system of devolved governance in Kenya, responsibility for disease surveillance, within the animal and human populations, lies with the 47 semi-autonomous counties as laid out in schedule 4 of The Constitution of Kenya 2010, while the national level retains policy making powers within the health and veterinary sector (19). Counties are under the governance of the County Assembly and the County Executive Committee; with county functions and services subsequently decentralised to the administrative unit of the sub-county under the office of the sub-county administrator as per section 50 of the 2012 County Governments Act (20). The ZDU provides epidemiological support and outbreak response for zoonotic diseases and has provided training for OH focal persons at the county level to encourage cross-sectoral collaboration within the devolved system (16).

Currently there is little documentation on the uptake of cross-sectoral collaboration within disease surveillance at the sub-national level. Understanding what the drivers and barriers are to adopting cross-disciplinary ways of working is an important step in designing strategies to enhance these practises and support potential future integration in surveillance, whilst bolstering the more general aspirations of the scientific community to rollout OH approaches.

We undertook the current study to better understand these drivers and barriers to cross-sectoral collaboration within the current disease surveillance systems at the sub-national level in a country with a stated aim to operationalise OH. The study forms part of the “ZooLinK” programme, which aimed to support the development of an integrated zoonotic disease surveillance system which may serve as a model for other counties in Kenya. We consider surveillance to encompass the systematic collection, analysis, and dissemination of disease data which explicitly contribute to mitigation actions (21). We consider integration to be the institutionalisation and formalisation of a spectrum of collaborative activities between the human and animal health sectors, from regular data sharing or joint disease response activities, to the adoption of a fully interoperable data collection, analysis and dissemination system potentially utilising shared diagnostic laboratories. The different aspects of collaborative processes within disease surveillance have been recently reviewed by Bordier et al. (22).

Whilst a truly OH approach includes integration with data from the environmental sector, for the purposes of this study only the human and animal health sectors were considered. The ZooLinK research programme itself utilised shared diagnostic and data facilities to facilitate dedicated animal and human surveillance teams to collect, analyse and disseminate data on 15 zoonoses of interest within sentinel sites in western Kenya including health care facilities, livestock markets and abattoirs as described in detail by Falzon et al. (23).

## Methods

The objective of the study was to identify themes relating to the barriers and drivers for the integration of animal and human health surveillance systems at the sub-national level. It focused on the 12 sub-counties covered by the “ZooLinK” surveillance activities (23), within the counties of Kakamega, Busia, and Bungoma in western Kenya where several zoonotic infections have been found to be co-endemic (24, 25). The counties of Kakamega, Busia, and Bungoma have populations of 1. 87 million, 0.89 million, and 0.99 million people respectively as of the 2019 population and housing census (26), with mixed crop-livestock smallholdings being the predominant farming system (25).

We conducted semi-structured interviews with surveillance officers across human and animal health sectors in the selected study sites. Semi-structured, key-informant interviews were chosen to allow for narratives to emerge and the ability for the conversation to flow, whilst being guided by questions which aimed to draw out the drivers and barriers to cross-sectoral collaboration. The interview guide can be found in Supplementary Material 1 but briefly, participants were asked to recall a time in which they were involved with a report of, or response to, a zoonotic disease event and this narrative along with probing questions, was used to tease out aspects of cross-sectoral collaboration and communication. Additional probing questions were included on the flow of information both vertically (from county to national level and back) and horizontally (between counties), the prioritisation process for surveillance activities and strengths and weaknesses of the surveillance systems in general, to gain a greater overview of the workings of the system.

**Figure 1:**
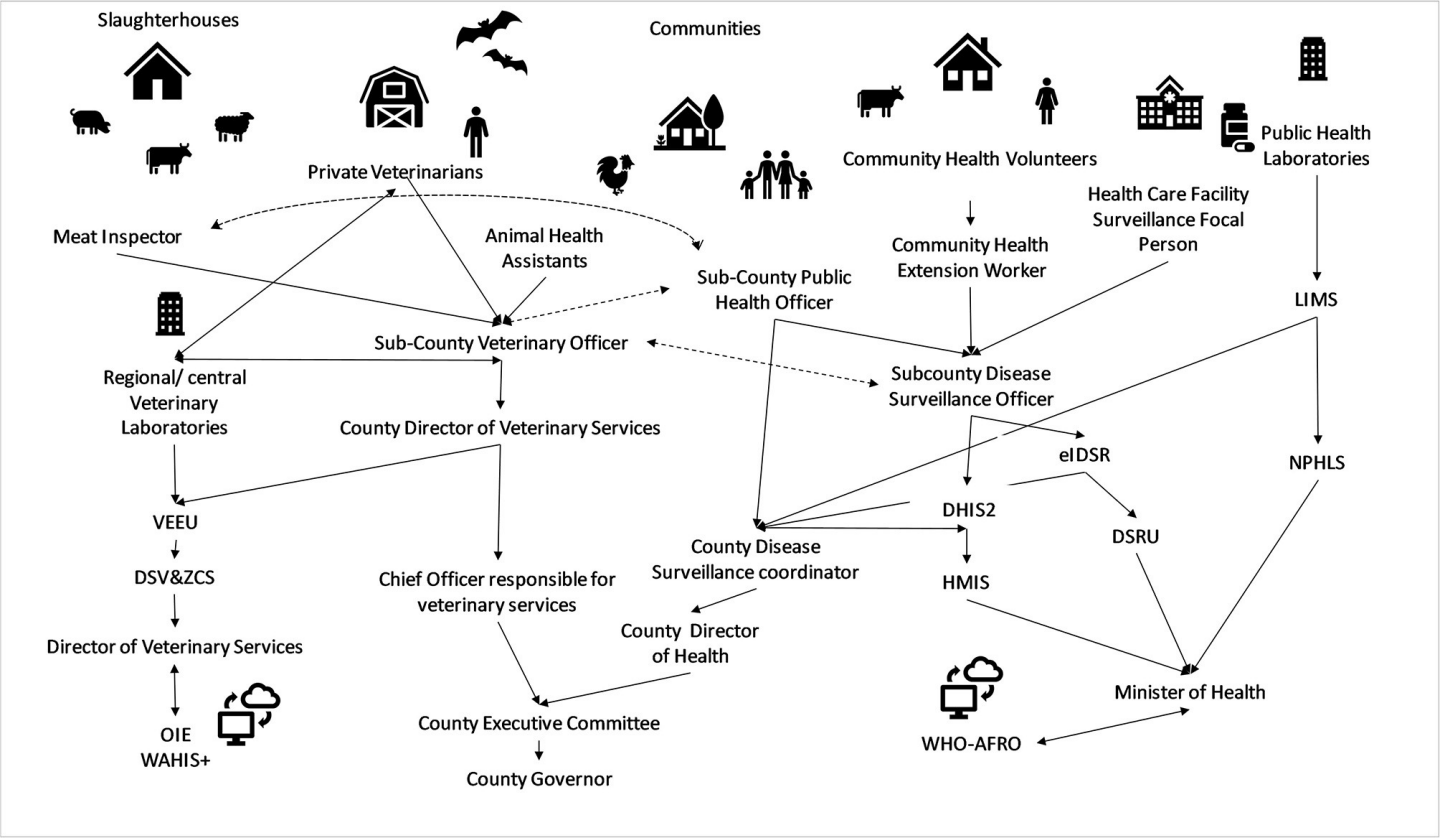
Simplified overview of animal and human disease surveillance information flow in Kenya. 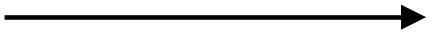 indicates flow of surveillance data through designated data collection tools. 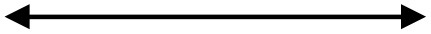 indicates data flow in both directions. 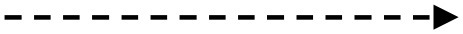 indicates lines of communication mandated by disease specific acts (i.e., Rabies Control Act, Meat Control Act) without specific reporting tools. DHIS2, District Health Information System 2, eIDSR, electronic Integrated Disease Surveillance & Response system, HMIS, Health Management information Service, DSRU, Disease Surveillance & Response Unit, LIMS, Laboratory Information Management System, NPHLS, National Public Health Laboratory Services, WHO-AFRO, World Health Organization Africa Region, VEEU, Veterinary Epidemiology & Economics Unit, DSV&ZCS, Disease Surveillance, Vectors & Zoological Control Services, OIE WAHIS+, World Animal Health Information System.

The study used purposive sampling, targeting government officers with direct responsibility for collecting, analysing and disseminating disease surveillance data. Figure 1 provides a simplified illustration of the current structure of animal and human disease surveillance in Kenya. The specific officers participating in this study and their roles are elaborated in Table 1. The initial contacts in each county were the County Director of Veterinary Services (CDVS) and the County Director for Health (CDH), who provided permission to conduct the study and in turn identified the appropriate officers at the county and sub-county level to participate in the interviews, with a total of 30 potential key informants. Our focus was the formal government surveillance system and did not extend to disseminated surveillance by the population, such as participatory disease surveillance systems whereby members of a community actively report disease events (27).

**Table 1:** Study participants and their roles and responsibilities within disease surveillance in Kenya.

**Role**	**Acronym**	**Responsibilities**	**Number participating**
County director veterinary services	CDVS	• Responsible for the management of veterinary services across the county including the organisation of surveillance and the planning and co-ordinating of disease control programs. • Receives and aggregates data from SCVOs and reports by email to the head of the Veterinary Epidemiology & Economics Unit (VEEU) who are responsible for analysis of data and onward reporting. • The CDVS also report to the county executive committee via the chief officer	3 (1 acting)
Sub-county veterinary officers	SCVO	• Implement veterinary services at the decentralised unit, including disease control activities and surveillance. • Received written or SMS reports from meat inspectors and animal health assistants collates and report to the CDVS.	12
County disease surveillance coordinators	CDSC	• Responsible for the planning, formulation and supervision of disease surveillance activities in the county. • The CDSC isIsis expected to analyse electronic Integrated Disease Surveilannce & Response (eIDSR) and District Health Information System 2 (DHIS2) data on a weekly basis • Reports and reports to the County Director of Health who in turn reports to the County Executive Committee	3
Sub-county public health officers	SCPHO	• Coordinate public health activities at the sub-county level including disease control services, inspect food processing and retail establishments and receive disease reports from community members. • Report, by phone or hard copy to the SCDSO or direct to the CDSC where a SCDSO is not in post	7
Sub-county disease surveillance officers	SCDSO	• Implements disease surveillance activities within the sub-county. • Obtains reports from health facility surveillance focal persons by SMS or hard copy, aggregates and uploads data onto eIDSR and DHIS2. • eIDSR and DHIS2 data can be viewed by the National level units—Health Management Information Systems (HMIS) & Disease Surveillance & Response Unit [(HMIS & (DSRU)].	4

The data collection took place over two periods of 2 weeks each in June and July 2018. Officers were initially contacted by phone to arrange a date and time for an interview and they were visited at their place of work. Those unavailable in the first data collection period were asked for a suitable appointment in the second data collection period.

Twenty-seven semi-structured interviews were conducted with a total of 28 veterinary and public health officers at the county and sub-county level who were available during the data collection period (two officers wished to be interviewed together). The participants and their roles within the surveillance system are described in Table 1.

Two of the SCVOs requested to be interviewed together, while all other participants were interviewed in a private space within their place of work in one-on-one interviews. Twenty-six participants were male and two were female (both within the human health sector), reflecting the gender disparity within the decentralised civil service of Kenya, particularly at managerial levels due to multiple structural barriers still present within many institutions (28).

After obtaining the written informed consent of the participants, interviews were conducted by the first author using an interview guide (Supplementary Material 1). The interviews lasted between 38 and 95 min, were audio-recorded and transcribed verbatim by the first author. The audio recordings were also supplemented by field notes, predominately noting particular sentences which jumped out during the interview and which were then used to direct some of the initial coding.

Thematic analysis, facilitated by the NVivo12^®^ software (QRS International) (29), was conducted predominately inductively, to determine the emergent themes and sub-themes salient to cross-sectoral collaboration at the sub-national level. NVivo12^®^ is a computer-assisted qualitative data analysis software which provides a user-friendly interface in which to code and sort textual data, a process which previously would be undertaken by highlighting or physically cutting out text and sorting it into groups (29).

After transcription, the transcripts were read several times to aid familiarity. After uploading the transcripts into NVivo12^®^, specific parts of the text relating to cross-sectoral collaboration were categorised under an initial set of codes. Codes are essentially labels which assign a related meaning to sections of text from different sources as illustrated in Table 2 (29). The codes were then grouped into an initial set of themes, the content of which were then further interrogated and re-grouped. This process was re-iterated several times, until what we believe to be an inclusive yet parsimonious set of themes were described and no further themes were emerging from the text.

**Table 2:** Code Book describing codes emerging from the transcripts and the themes under which they were grouped.

**Overarching theme**	**Code(s)**	**Description**
1.0_ Common objectives	1.1_Issues for action	Used to capture comments that talk of communication or actions taken with other ministry on specific topics
	1.2_Examples of action	Used to capture comments that describe the type of action taken and frequency thereof
2.0_ Operational structures	2.1_Legislation & targets	Used to capture comments that describe legislation relevant to surveillance activities
	2.2._Hierarchies & protocols	Used to capture comments describing relationships between actors and the protocols or hierarchies which govern those relationships
	2.3_Data sharing	Used to capture comments on the mechanisms by which data could be shared, both within each sector and between sectors
3.0_Appropriate resources	3.1_Resources	Used to capture comments that describe the concerns of actors regarding the presence and absence of resources (financial, infrastructural and human) required to do their job
4.0_Political will	4.1_Political_interests	Used to capture comments regarding national and sub-national interests and priorities and the drivers of these including pressure/interests from voters
	4.2_External interests	Used to capture comments regarding international (Inc. international organisations, donors etc.) interests and pressures

### Ethical Approval

Approval was granted by the Institutional Research Ethics Committee (IREC Reference No. 2017-08) at the International Livestock Research Institute, a review body approved by the Kenyan National Commission for Science, Technology and Innovation. Approval to conduct the work was also obtained from the Ministry of Agriculture & Irrigation—Directorate of Veterinary Services and the Ministry of Health, and the relevant offices of these Ministries at devolved government level.

## Findings

Through the process of thematic analysis, we have identified four themes relating to the drivers or the barriers of cross-sectoral collaboration. We have classified these themes as; “Common Objectives,” “Operational Structures,” “Appropriate Resources,” and “Political Will.”

### Common Objectives as a Driver of Cross-Sectoral Collaboration in Disease Surveillance

Participants were asked to describe situations in which they had communicated, or carried out joint activities, with their counterparts in the opposite ministry. The majority of participants were able to give examples of cross-sectoral communication and collaboration already taking place with others expressing a desire for a more integrated approach.

“*If I could advise the national government, this department of public health and the department of veterinary services, they should have at least one unit, if they were brought under one unit and came under one department so that at least these people work as a team*” [MoH7]

Participants have different experiences of integration, reflecting, we believe the somewhat *ad hoc* nature of cross-sectoral collaboration at this time. This is illustrated by the differing reflections of two participants where a surveillance officer from the veterinary sector uses an informal approach to keep lines of communication open, and the officer from human health has experienced a reactive system which comes to life when needed.

“*Interaction* [between sectors]*, more or less on a daily basis, it can be formal or informal […] but there is a lot of transmission of data”* [MALF2]“*This system* [of One Health] *is weak, but it becomes active if we have an outbreak […] It depends on the situation, when things are calm the links are down but when we have outbreaks we receive communications and share information”* [MoH5]

Details were requested on the focus of interactions which the participants had experienced. These interactions focused on a handful of issues which were consistently highlighted, being response to dog-bite events or suspected rabies cases, carrying out meat hygiene related duties, and responding to potential anthrax cases.

“*Yes, actually that's one of the main things we work with public health on […] they* [those bitten by dogs] *end up in our office to find out if the dog was vaccinated or not, we have been working closely with public health on dog bites”* [MoA9]“*We also have meat products, we do surveillance of products, our colleagues* [in the DVS] *inspect meat at the slaughterhouse and when it reaches the butchery we come in, we monitor at the butcheries and if we hear from the community that ‘so and so was bringing meat in a sack' we follow-up […] so that is how we collaborate with the veterinary department”* [MoH6]

The nature of the cross-sectoral collaborations reported to us by participants were predominately reactive (to dog-bite cases) rather than pro-active (vaccination, dog management and community sensitisation). We have designated these issues as “*common objectives*” and see them as the most proximal driver of cross-sectoral collaboration. The particular examples of common objectives appear to be closely linked to the available “operational structures” we identified from our study, which are discussed in the following section.

### Operational Structures as Drivers or Barriers for Cross-Sectoral Collaboration in Disease Surveillance

Under “operational structures” we grouped issues which arose pertaining to the legislation guiding the work of disease surveillance within each sector, the hierarchies and protocols which guide the interaction of the officers, and data sharing platforms or protocols. The presence of these factors is a driver to action on “common objectives,” while absence of any one factor becomes a barrier.

### Regulatory Environment

The need to have a supportive regulatory environment came out clearly in providing a structure within which officers from the two ministries can work. The response to suspected rabies cases and meat hygiene were seen to be facilitated by the clear demarcation of responsibilities, enshrined in legislation under the Rabies Act (Cap 365), Animal Diseases Act (Cap 364), Meat Control Act (Cap 356), and the Food, Drugs & Chemical Substances Act (Cap 254). These pieces of legislation provide officers from the veterinary services and Ministry of Health, respectively, the authority to act in a co-ordinated manner within differing parts of the system.

“*We have the animal diseases act which gives* [officers] *the mandate to carry out any inspections […] The meat control act specifically addresses the issues of what is consumable or not”* [MALF10]

“*If we have cases here of dog bites they are assessed by the clinical officer, then we will advise on the anti-rabies vaccination […] at that point we will liaise with the veterinary officer to take action on the dogs”* [MoH10]

### Formal Lines of Communication

Observation of existing hierarchies and protocols were seen as an important factor in enabling communication of officers between sectors and it was generally felt that enhancing collaboration would require new formal structures for communication and data sharing.

“*[…] these things need to be structured, I cannot walk in and say the DVS has sent me here to discuss disease […]”* [MALF4]

One county has a OH focal person, a veterinary officer, in office, providing a formalised route for cross-sectoral collaboration. Within this county, adhering to formal lines of communication was identified as being a cornerstone for success of the initiative.

“*We have been having meetings under the One Health office […] it's not just in passing, it's a formal way of interacting.”* [MALF11]

While the formation of County OH units is a stated priority, they are yet to be implemented across every county. Where such formal structures are not yet in place there is a reliance on personal relationships between the surveillance officers in different sectors to facilitate informal collaborative networks. Such informal networks do not lend themselves to building institutional memory and may be lost as staff retire or move on.

“*…they* [communications] *tend to be more personal*, [depending on] *which officers are holding the office. Once there are good relations, it goes down to the other staff”* [MALF10]

Devolution, whilst allowing innovative solutions to complex health and veterinary problems to be formulated at a local level, was identified by participants as leading to further complexities within disease surveillance providing the potential to slow the transmission of data between sub-national level and across county borders.

“*It's like we* [the counties] *are now different groups, we rarely interact*” [MALF8]

### Data Sharing

In addition to formal channels of communication there is a need for effective data sharing between sectors. There is currently no interoperable data sharing platform for human and animal health data sharing at the sub-national or indeed national level. Participants also spoke of difficulties in data sharing within their own sectors which must be addressed to ensure timely, accurate flow of data from the sub-national level to the national. Appropriate feedback to the sub-national level was identified as being of significant importance to the action of disease surveillance. A lack of such feedback, even in the form of negative consequence for non-reporting, was cited across both sectors as a disincentive to reporting, leading to demotivation of officers.

“*Disease reporting in the county is almost dead, because you know when you report on this and this situation you also expect feedback and when there is no positive feedback people get wearied out and then stop. Because even when you reprimand they say, last time you did nothing, why should I waste my energy?”* [MALF10]“*I think* [the data is used] *at county level and national, I'm not sure… they* [the national ministry] *just keep information [.] we used to have quarterly data review meetings, but last year they stopped happening”* [MoH11]

### Appropriate Resources as a Driver or Barrier for Cross-Sectoral Collaboration in Disease Surveillance

The operational structures which facilitate cross-sectoral collaboration on common objectives require appropriate resources at all levels. Participants in our study identified aspects of financial, human and infrastructure resources as currently hindering their disease surveillance activities and ability to collaborate across sectors.

“*What blocks me most is lack of personnel to do the work, the second is resources, financial, transport, movement …we don't have a laboratory e.g., so whenever you have a case, you just do it by clinical diagnoses, you can't confirm and say ‘this was rabies, this was anthrax'.”* [MALF9]“* The challenges are financial constraints. We are not able as one health, to attend meetings, we are 2 ministries so bringing people together requires resources”* [MoH3]

To achieve the resource commitments needed requires decision makers in charge of resources to have the will for a change; this is explored in the next section.

### Political Will as a Driver or Barrier for Cross-Sectoral Collaboration in Disease Surveillance

Participants identified several different parties who had an influence on the operation of disease surveillance at the sub-national level. These interests were identified as being related to setting priorities and subsequent resource allocation to surveillance activities.

Particularly relevant to zoonotic disease surveillance, we noted a disparity in the prioritisation of zoonotic diseases between the animal and human health sectors. Zoonotic diseases were cited by all participants from animal health as being amongst their priority diseases, with rabies (15 respondents) and anthrax (13 respondents) being most common. Within human health however, only one participant felt that zoonoses (anthrax and brucellosis) were a local priority. Interestingly, this officer was located in a county with an active OH focal person. Participants reported that priorities are set at the county level, but are often aligned with the national targets.

“*They* [the county priorities] *are the diseases stipulated at the national level for eradication: that is AFP* [Acute Flaccid Paralysis], *measles […] As a county we* [also] *have conditions, maternal death, malaria, that are the diseases of priority”* [MoH11]

Participants were aware that prioritisation at international, national and sub-national level is needed to ensure that appropriate resources are provided to surveillance and disease control activities, with participants perceiving a particular lack of interest in surveillance from budget holders at the sub-national level. The priorities of technical staff were seen to be subsumed by the priorities of the elected members of the county assemblies and the political appointees within the county executive committees (CEC). The CECs have control of the budgetary allocations with participants believing that they prioritise “curative” health care or “visible” investments such as agriculture inputs (fertilisers etc.) over surveillance activities.

“*The people at the top, they don't consider surveillance as a priority […] they prioritise purchase of inputs, fertilisers, many millions on fertilisers, tractors, to give an impression to farmers that resources are close to them.”* [MALF11]

Some participants perceived that momentum may be growing at the national level, but were sceptical that this interest was likely to be translated into resource allocation at the sub-national level.

“*The top brass were in a seminar in Kisumu and emphasis was put on putting some resources on the prevention and control of zoonotic diseases. How successful that was remains to be seen as all the county governments have their own priorities. You can budget for anything but the county assemblies divert it for some other use”* [MoH3]

Participants were also very aware of the re-enforcing cycle of political will, where lack of funding leads to lack of data leads to lack of political will *Ad infinitum*.

“*Because no one is funding it, no one is questioning, no one wants to know what happens in surveillance. The county sees health as treating, it doesn't see health as preventing and informing, so it doesn't actually see that there is a need for surveillance. It* [the county] *sees surveillance as an item that is eating the money without giving back. They would rather see that we buy medicine, equip our hospital.”* [MoH4]

National and county priorities were seen to be influenced in turn by those of the international community and other external funders. External funders were acknowledged by participants to come with their own specific interests. WHO and the “Global Fund” (to fight AIDs, TB and malaria) were among those external bodies who were identified to drive the health agenda, and the potential for such external support to enhance the surveillance of zoonoses was discussed.

“*Generally we have a problem when it comes to surveillance, it's not like those diseases, AIDS, TB and malaria funded directly by the Global Fund. But for surveillance, if we get a sponsor to support us we could be able to manage those* [zoonotic] *diseases very well”* [MoH7]

International interest in Avian Influenza (AI) had previously led to the formation and training of rapid response teams. A subsequent scare in neighbouring Uganda galvanised local response, demonstrating the potential for local and international interests to converge and provide appropriate support for OH.

“*We were trained sometime in 2014 on AI, how to detect, how to respond, how to form a rapid response team. When there was a scare last year in Uganda, we communicated, and we prepared ourselves, we were on the alert, we talked to the public health officer, the nursing officer, the Deputy County Commissioners and even the police…we prepared to handle any eventualities but luckily enough there were no cases”* [MoA5]

The interest in such events, while important in galvanising collaboration, has however, the potential to be transitory, with the potential that technical officers are pulled from one activity to the next as focus of politicians shifted.

“*I think maybe what happened is the scare somehow faded, because when there was the first scare, county leadership really wanted to know what was happening. It's* [Avian Influenza] *on the news everywhere and we tried to take some steps, but then the information slowly faded so I think they* [politicians] *forgot about it.”* [MoA8]

## Discussion

In our study we identified four key themes; “common objectives,” “operational structures,” “appropriate resources,” and “political will” which were related to the drivers and barriers for cross-sectoral collaboration by disease surveillance officers at the sub-national level. The most proximal driver for collaborative actions between sectors are “common objectives.” Action on these common objectives is facilitated by the presence of “operational structures” such as specific legislation, clear reporting protocols and interoperable data sharing systems. Setting up and working within these structures requires “appropriate resources” including finance, human resource and physical infrastructure such as laboratories, vehicles, IT and consumables. The allocation of such resources is driven by “political will” at the international, national and sub-national level, with this political will and the resource and structures which flow from it in turn influencing the common objectives, the pursuit of which drives action.

**Figure 2:**
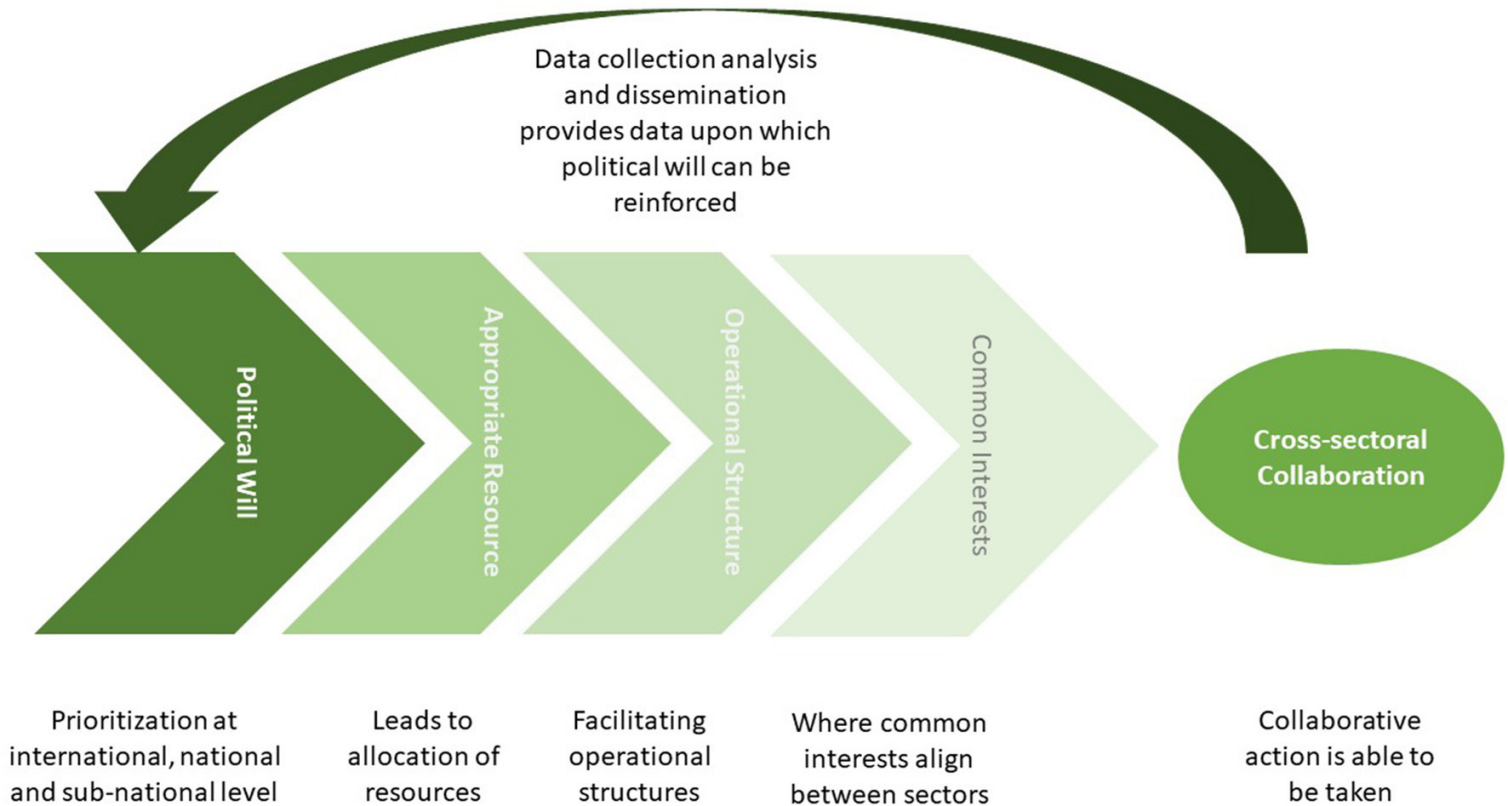
Conceptual framework of how identified themes enable cross-sectoral collaboration within disease surveillance activities.

These themes sit within a framework which can be visualised as a “hierarchy of needs” with a self-reinforcing feedback loop, as illustrated in Figure 2. We see the themes interacting in a sequential way in which each theme becomes a facilitator of the next. In this way the presence of political will allows the allocation of appropriate resources, facilitating the operational structures within which action can be taken on areas in which the objectives of each sector align. A feedback loop then exists where once disease surveillance data are collected, analysed or disseminated in a cross-sectoral manner, the data themselves may reinforce the political will upon which the drivers of collaboration are built. The absence of any one of these identified themes acts as a barrier to the successful implementation of cross-sectoral collaboration within disease surveillance.

We had anticipated that the alignment of disease surveillance priorities in the human and animal health sectors would be a driver for “common objectives,” yet the common objectives we identified were not necessarily aligned to the sector priorities as stated. Animal health officers prioritised zoonoses, particularly anthrax and rabies, as per a prioritisation exercise driven by the GHSA (15). Diseases identified as being priorities for surveillance within human health were very closely aligned to those described within the Integrated Disease Surveillance & Response framework standard case definitions for priority diseases in Kenya and were predominately non-zoonotic.

Acute flaccid paralysis as a syndrome indicative of poliomyelitis was mentioned by all human health surveillance officers interviewed as being a surveillance priority reflecting the influence of priorities set by international targets. Poliomyelitis has been earmarked for eradication through the Global Polio Eradication Initiative (GPEI). GPEI is now embarking upon the “endgame” strategy 2019–2023 but until eradication is achieved, there remains a risk to poliovirus free-countries and as such it was declared a Public Health Emergency of International Concern within the International Health Regulations 2014.

Our interpretation of the data is that at the sub-national level the “common objectives” are currently driven primarily through the presence of “operational structures,” specifically legislation. Specific pieces of legislation were recognised by participants as providing clearly demarcated responsibilities for officers such as the Animal Diseases Act (Cap 364), Meat Control Act (Cap 356), the Food, Drugs & Chemical Substances Act (Cap 254) and the Rabies Act (Cap 365). These acts provide clearly demarcated roles to actors from both veterinary and public health sectors, implicitly recognising the interconnectedness of human and animal health in relation to zoonoses and food safety, despite not being explicitly built upon OH principles. The presence of this legislation indicates that “common objectives” have, at the point of legislating, been prioritised at one or more of the policy making levels (international, national or sub-national).

We suggest therefore, that legislative frameworks are a powerful driver of collaborative surveillance and the existence of legislation is in itself an indication of the presence of political will at one of the policy making levels (international, national or sub-national). The importance of appropriate legislation, which clearly demarcates roles and responsibilities to allow cross-sectoral collaboration has been highlighted in a recent global review of integrated surveillance systems by Bordier et al. (22).

Participants in this study identified formalised mechanisms of communication and data sharing between the human and animal health sectors at the sub-national level and between the sub-national and national levels as a key requirement for effective service delivery. These issues have previously been highlighted in reports from two evaluations conducted in 2017 by the WHO and the FAO. The joint external evaluation evaluated the IHR capacity of Kenya, whilst the FAO Surveillance Evaluation Tool (SET) evaluated animal disease surveillance including zoonoses (30, 31). Both reports commended Kenya on its leadership in the implementation of cross-sectoral integration through the ZDU, although both evaluations identified specific weaknesses relating to the lack of formalised communication forums and lack of systematic data sharing between sectors, particularly at the sub-national level.

The formal integration of data streams currently collected within distinct, highly vertical structures governed by differing pieces of legislation is a complex challenge to address. Several examples are available of the integration of human and animal health data within a unified system (22, 23, 32, 33) yet it is important that any system implemented fits within existing structures without duplication of effort, has the appropriate legal basis regarding data ownership, confidentiality etc., and appropriate resources to facilitate its implementation.

An aspect of data sharing which was not raised by participants, biased as this study was toward public sector stakeholders, is the additional need for appropriate data sharing between the public and private sectors. The collection of disease surveillance data by private sector actors is of particular interest within the veterinary sector, where private veterinarians are often the front-line service providers and where agri-food businesses may regularly collect data for their own internal management practises.

Private veterinarians in Kenya are currently mandated to report only notifiable diseases under the Animal Diseases Act (Cap 364). Despite the privatisation of veterinary services across sub-Saharan Africa following the structural adjustment policies of the 1980's, the private veterinary sector in Kenya is still outweighed by the public sector, particularly outside of urban, and highly productive agricultural zones. Approximately 1/3 of all veterinarians in the country are currently within the private sector, but approximately 95% of the data reported into the surveillance system come from public sector actors, indicating a potential ongoing bias in reporting to the detriment of the national surveillance performance (Dr. Kahriri, VEEU, *Per. Comms*.).

Strengthening the participation of the private sector in disease surveillance activities was a key recommendation of the 2011 OIE PVS mission (34). As the commercialisation of agriculture continues across East Africa with the formation of larger, more industrial agri-businesses, these public-private linkages within disease surveillance will become ever more important, including with private veterinary para-professionals whose services often dominate in pastoral areas (35). There are examples where private sector data have been successfully integrated into publicly funded surveillance platforms (36). It is important however, that full consideration is given to the basis on which such data sharing occurs which may require a legislative framework, covering data ownership and data use.

An appropriate financial model which facilitates the integration and use of data collected across animal and human health, as well as recognising the benefits accrued across the public and private sectors, is urgently needed and the allocation of resources to surveillance was a ubiquitous theme raised by participants in this study. As the implementation of disease control and surveillance is now the responsibility of the devolved governments, the process of prioritisation and building of political will at the sub-national level is crucial in order that appropriate allocation of resources is achieved. In accordance with findings in other counties, participants in our study perceived the priorities of county governments to be agricultural inputs (fertilizers, seed) or “curative” health services, both of which may potentially be more visible to the electorate than issues of disease surveillance. Surveillance systems within both sectors are therefore under considerable resource constraints to fulfil their current mandate.

Stimulating investment in surveillance activities in general, and cross-sectoral collaboration specifically, must be done within the context of competing priorities within constrained public expenditure on health and agriculture, both of which currently fall below the internationally agreed targets. The 2003 Maputo Declaration stipulated that countries should allocate 10% of public expenditure on Agriculture, while the 2001 Abuja Declaration set a target of 15% of public expenditure to be allocated to health (37, 38). Between 2013 and 2017, public health expenditure on health in Kenya averaged 6.4% of total government spending, with public agricultural expenditure averaging 5.5% over the same period.

At the sub-national level, counties in Kenya are predominately reliant on an equitable share of nationally raised revenue (84%) in combination with conditional grants (5%) and locally raised revenue (11%). Absorption rates of the county governments from the national allocated budgets have slowly increased but remain low, with an average absorption rate of 65% in 2016/17. Budgetary absorption indicates the ability of the counties to spend the budgetary allocation and is positively associated with efficient and effective budgetary management, which in turn relies upon strong capacity within the county finance departments (39). Local revenue collection also lags behind projections with only 22 counties in 2016/17 achieving an average 60% of their revenue collection targets (40).

It is within this context of resource constraint that new mandates, such as cross-sectoral collaborative structures, must make a business case to the CECs and the county assemblies, who are responsible for budgetary allocation at the sub-national level.

Initial investment in the operational structures required for the implementation of integrated surveillance programmes may be beyond the reach of national and particularly sub-national budgets, and this is an area in which external catalytic funding may play a role (41). The national rabies elimination strategy (NRES) acknowledges that current funding from the ministries is insufficient and that a variety of funding sources, including external donors, is required for implementation.

There is currently an explicit expectation by the ZDU that external support will be required to fully operationalise its' mandate at the sub-national level (16). Reliance on external donors, however, must be undertaken with caution as it has the potential to undermine the national or sub-national strategic priorities. Analysis by the World Bank suggests that approximately 1/3 of health expenditure in Kenya is via donor spending, the majority of which is not aligned to government priorities (42). A review of global public health expenditure by the WHO re-iterated this disconnect, demonstrating that 46% of all donor funding for health is channelled to HIV/AIDS, TB and malaria, but that this funding does not directly correlate with either national prevalence levels of these diseases or the GDP per capita of the countries receiving external funding (43).

In the context of animal and human health systems which already lack sufficient public funding, it is important that any drive to strengthen cross-sectoral collaboration at the sub-national level is done in a way which does not detract from operation of the underlying systems, but rather actively strengthens them (44, 45). It is also important that any systems used for cross-sectoral communication and data sharing do not add to an already confusing surveillance structure, particularly within the animal health sector where numerous surveillance tools are currently being utilised in an un-coordinated manner (30).

At a national and sub-national level, stimulating investment for cross-sectoral activities will require incorporating the concept within the key strategy documents for the “parent” ministries. Currently, neither the Kenya Health Policy 2014–2030, nor the Agriculture Sector and Development Strategy 2010–2020, nor the National Agriculture Investment Plan 2019–2024 explicitly reference such activities in the context of infectious disease (46, 47). Similar omissions are made in the integrated county development plans of Busia, Bungoma and Kakamega counties (48–50).

It would be useful to build upon the data collected through our study with the perspectives of those working in political positions to better understand their resource allocation decisions. Several counties are in the process of bringing county level legislation into law for public health and animal health (Dr. Ogendo CDVS Busia County, *Per. Comms*.). The process of formulating county-level legislation not only clarifies the counties' position in post-devolution Kenya but also provides an opportunity to ensure legislation is fit for purpose where remnants of colonial era legislation still exist. It will be interesting to observe if this enhances the agency of the county governments to improve resource allocation to disease surveillance. Conversely, county-specific legislation may further fragment an already decentralised disease surveillance system and result in slower response to diseases which occur across county boundaries.

Allocation of resources at the sub-national level will also be guided by the evidence of the cost-effectiveness of surveillance and of cross-sectoral collaboration, as exemplified by the statement “*It* [the county] *sees surveillance as an item that is eating the money without giving back.”* More robust surveillance data collection systems and importantly the utilisation of that data is needed to inform economic analyses both for “traditional” and “integrated” surveillance systems. Little empirical data are yet available on the cost-effectiveness of integrated systems. Furthermore, novel cost-sharing structures are required to ensure that costs are correctly attributed across sectors in proportion to where benefits are accrued, as illustrated by the proposals for cost-sharing in relation to brucellosis vaccination in Mongolia (51).

Spending budget lines across differing ministries may be challenging and therefore cost-sharing scenarios may also require novel financing modalities, such as a dedicated shared budget envelope for the surveillance and control of zoonotic diseases. In Kenya, the existence of the ZDU may facilitate such an innovation, yet the concern is that this may result in a dedicated zoonoses surveillance system running in parallel to the “core” business of the 2 ministries, rather than encouraging truly collaborative or integrated working.

A greater understanding of the correct attribution of costs and benefits of OH interventions could conceivably allow for allocations made to one sector (i.e., veterinary services) to be counted against public expenditure targets in another (i.e., human health), if the expenditure can be empirically associated with benefits in the latter sector. This may allow for appropriate resource reallocation while allowing countries to reach their targets for public expenditure, such as those set through the Abuja & Maputo declarations (37, 38).

Conceptual frameworks have been constructed to assess the cost-effectiveness of integrated surveillance which include the need to provide evidence of the intangible benefits of working in a collaborative manner (1). Several intangible benefits of OH working have previously been identified and may include; an increase in social and professional capital for the surveillance officers through expansion of their networks and technical capacities, improved professional opportunities, improved trust between sectors and an increased peace-of-mind for officers who can base their risk assessments and actions upon a greater pool of data (5, 52). The collection, analysis and dissemination of high-quality surveillance data provides a reinforcing loop in the identified themes, being a conduit to building the political will upon which the other themes stand.

The four themes which emerged from this study as being key facilitators of cross-sectoral collaboration within disease surveillance have synergies with some of the organisational criteria identified by Bordier et al. through which “OH” surveillance systems may be evaluated. The need for relevant common objectives, a range of vital operational structures, and the need for appropriate resources, was identified as being fundamental aspects of a functional collaborative system (53). Evaluation frameworks such as that proposed by Bordier et al. (53) and the Network for Evaluation of One Health (NEOH) (54) will be increasingly useful as OH continues to be operationalised in different contexts. The integration of these tools with new initiatives such as the IHR-PVS bridging workshops (55) would be a useful step to support countries wishing to advance both their sector specific and cross-sectoral goals.

The current study wished to understand the perceptions of disease surveillance officers within three counties of western Kenya on the barriers and drivers for cross-sectoral collaboration. The breadth of perspectives was limited and currently exclude those of politically appointed officers and frontline workers, including those in the private sector. It would be useful to elaborate on the current study and triangulating the themes identified here by working with a wider range of stakeholders, potentially across a wider geographical range.

Interviews for this study were conducted individually and therefore could not produce a combined consensus on issues. Focus group discussions or stakeholder workshops may have helped to produce such a consensus, though the information from this study provides a good basis on perspectives which future studies may build on. This study was conducted by a research team who have worked within the arena of “One Health” for many years and we acknowledge our potential bias in viewing cross-sectoral collaboration as a good to be maximised, based also upon the stance of both the national and international community.

Overall, the data analysis indicates constraint to developing and sustaining collaborative effort for integrated surveillance. There are some elements of collaboration which appear to work, but largely the institutional environment (the rules and their enforcement) does not encourage systematic collaborative practices. Due to this weak institutional environment, the allocation of resources to such activities has not been embedded in the system. Additionally, the element of prioritising diseases and health problems at a local level appears to be poorly institutionalised and draws predominately on national or even international priorities. Strengthening local prioritisation of health issues will require a focus on quantification of burden through robust surveillance data, along with the identification of key mitigation activities. In this way it would be easier to better evaluate the ability of integrated surveillance to yield net benefits to public health, and in turn stimulate further investment in such.

## Conclusion

Our study comprised in-depth interviews with disease surveillance officers from the human and animal health sectors within three counties of western Kenya. These in-depth narratives shed light on the perceptions of the barriers and drivers of cross-sectoral surveillance activities. The themes we identified emerging from these interviews relate to a pathway where collaborative activities occur in response to “common objectives” facilitated by the availability of “operational structures” and “appropriate resources,” in turn driven by “political will.” The absence of any one of these themes would become a barrier to operationalising cross-sectoral collaboration and we suggest that the pathway becomes self-reinforcing where the collection, analysis and dissemination of surveillance data can in turn strengthen political will.

We suggest that sub-national governments, both in Kenya and beyond, should be engaged to determine what resource allocation can realistically be achieved for disease surveillance, and supported to make allocation decisions based upon robust empirical data on disease burden and economic analysis. The common objectives identified: responding to rabies and anthrax cases and safeguarding meat hygiene, that currently drive cross-sectoral communication and collaboration could be embraced as entry-points to improve the integration of animal and human health surveillance in Kenya. The epidemiological and economic data generated through a strengthened disease surveillance system with appropriate mechanisms for cross-sectoral collaboration, communication and data sharing must then be analysed and disseminated to provide continued stimulus for investment.

## Data Availability Statement

The raw data supporting the conclusions of this article will be made available by the authors, without undue reservation.

## Ethics Statement

The studies involving human participants were reviewed and approved by Institutional Research Ethics Committee (IREC Reference No. 2017-08) at the International Livestock Research Institute. The patients/participants provided their written informed consent to participate in this study.

## Author Contributions

LT conducted the key informant interviews, transcribed recordings, undertook the thematic analysis, and produced a first draft of the manuscript. SB, LF, and OH supported the analysis and writing. EF and JR obtained funding, contributed to the conceptualisation of the study, and reviewed the manuscript. All authors read and approved the final draft of the manuscript.

## Conflict of Interest

The authors declare that the research was conducted in the absence of any commercial or financial relationships that could be construed as a potential conflict of interest.

## Supplementary Material

Supplementary Material 1 Interview guide.
