# Supplementary material for: Cross-Sectoral Zoonotic Disease Surveillance in Western Kenya: Identifying Drivers and Barriers Within a Resource Constrained Setting
Source: Front Vet Sci. 2021 Jun 8;8:658454. doi: 10.3389/fvets.2021.658454 (PMC8217437; doi:10.3389/fvets.2021.658454)
Supplement: Supplementary Material 1 — Interview guide. [file Data_Sheet_1.PDF]

Key informant interviews

Introduce myself & the ZooLinK project

Request informed consent & detail confidentiality policy and request permission to record the interview for transcription at a later date.

Unique identifier:

1. Have you been involved in a response to a zoonotic disease outbreak report over the last 12 months? (if not then any disease outbreak) If so are you able to talk me through the timeline of reports and responses and indicate the resources required to mount this response?

   If the respondent talks about a zoonotic disease:

   Did you work with any colleagues from the veterinary/health [as appropriate] department?

   Did you have any communication with the Zoonotic Disease Unit?

   What were the challenges in working inter-sectorally?

   What were the advantages in working together?

   Would you class this response as a success?

   Is there anything you would have done differently?

   Aim here is to try and understand how the surveillance system already mapped works in reality and how this translates to action. I hope I will un-earth how people relate to each other, what resources are used etc If communication issues arise during this discussion then these will be probed as per 3,4,5 below

If no zoonotic disease discussed above then move to no.2

2. Have you been involved in activities related to H5N1 HPAI surveillance? If so are you able to describe this activity

   When was this?

   What activities were put in place for this surveillance?

   Where did the motivation for this surveillance activity come from? (eg international priorities, national priorities, county priorities)

   Did/do you work with any colleagues from the veterinary/health [as appropriate department?

Did/do you have any contact with the Zoonotic Disease Unit?

What were the challenges/benefits of working cross-sectorally?

Can you envisage such a system being used for other diseases?

Are there data captured on the different aspects of costs for the AI surveillance?

Is there any analysis of the cost-effectiveness of the surveillance? Yes/No

If Yes, what outcome measures are used for the cost-effectiveness analysis?

This question wishes to understand what activities have been implemented for a zoonotic disease. Again communication issues as per 3,4,5 will be probed as they arise

3 – 5 to be asked if not already covered above

3. In your opinion how well is surveillance data communicated between the county and national level?: Good/ adequate/ insufficient/ bad/ non-existent/ other

   Would you be happy to explain your answer?

   Have you any ideas how this may be improved?

   It has been suggested that the flow of information is being blocked between county and national due to devolution. I am interested in hearing the general opinion on that and how it can be rectified

4. In your opinion how well is surveillance data communicated between the national and county level? Good/ adequate/ insufficient/ bad/ non-existent/ other

   Would you be happy to explain your answer?
   Probe: Do you often receive feedback from national level after disease reports have been made?
   Do you feel that you receive feedback on how well surveillance is being performed?

   Have you any ideas how this may be improved?

5. In your opinion how well is surveillance data communicated between counties

   Would you be happy to explain your answer?

   Have you any ideas how this may be improved?

6. Do you believe that national and international surveillance priorities are well communicated to the county level? Yes/No

Are you happy to explain your answer?

7. Who or what do you consider guides your priority work areas in your county? (eg national/international legislation, policy, county governor, NGOs/donors/international bodies)

Trying to understand who makes decisions regarding resource allocation – and therefore who should be targeted to adjust the resource allocation to zoonotic diseases

If national policy legislation for zoonoses not already brought up above question 7 will be asked

<u>Do you know how these priorities are established? If so could you describe them.</u>

8. Do you consider the current national policy and legislature to have any relevance to the surveillance of zoonotic diseases at county level? Yes/No

   Would you be able to explain your answer?

I have a legislative framework for zoonoses – I wish to check if the policies/legislation I have identified really guides work on a day to day basis

9. Which diseases do you believe are a national priority?
   why do you believe that?

10. Which diseases do you believe are a priority at the county level?

    Why do you believe this?

11. Are there any differences in how you view your role in surveillance of zoonotic and non-zoonotic pathogens?

    I would like to identify If any of the key informants discuss inter-sectoral communication, this may have been brought up in questions 1 & 2

12. What do you consider to be the key strengths of disease surveillance in your county?

    Why do you think this?

13. What do you consider to be the key weaknesses of disease surveillance in your county?

    Why?

Thank you very much for your time today. If you have any questions about ZooLinK please call Dr Laura Falzon on xxxxxx for general enquiries or Dr Lian Thomas on xxxxx for questions relating to todays discussion.
